# MicroRNAs Regulating Renin–Angiotensin–Aldosterone System, Sympathetic Nervous System and Left Ventricular Hypertrophy in Systemic Arterial Hypertension

**DOI:** 10.3390/biom11121771

**Published:** 2021-11-26

**Authors:** Alex Cleber Improta-Caria, Marcela Gordilho Aras, Luca Nascimento, Ricardo Augusto Leoni De Sousa, Roque Aras-Júnior, Bruno Solano de Freitas Souza

**Affiliations:** 1 Post-Graduate Program in Medicine and Health, Faculty of Medicine, Federal University of Bahia, Salvador 40110-100, Brazil; roque.aras@uol.com.br; 2 Department of Physical Education in Cardiology of the State of Bahia, Brazilian Society of Cardiology, Salvador 41170-130, Brazil; 3 Center for Biotechnology and Cell Therapy, São Rafael Hospital, Salvador 41253-190, Brazil; 4 Faculty of Medicine, Federal University of Bahia, Salvador 40110-100, Brazil; marcelagaras@gmail.com (M.G.A.); luca.nascimento97@gmail.com (L.N.); 5 Neuroscience and Exercise Group, UFVJM, Diamantina 39000-000, Brazil; ricardoaugustoleonidesousa@gmail.com; 6 D’Or Institute for Research and Education (IDOR), Salvador 22281-100, Brazil; 7 Gonçalo Moniz Institute, Oswaldo Cruz Foundation (FIOCRUZ), Salvador 40296-710, Brazil

**Keywords:** microRNAs, hypertension, renin–angiotensin–aldosterone system, sympathetic nervous system, left ventricular hypertrophy

## Abstract

MicroRNAs are small non-coding RNAs that regulate gene and protein expression. MicroRNAs also regulate several cellular processes such as proliferation, differentiation, cell cycle, apoptosis, among others. In this context, they play important roles in the human body and in the pathogenesis of diseases such as cancer, diabetes, obesity and hypertension. In hypertension, microRNAs act on the renin–angiotensin–aldosterone system, sympathetic nervous system and left ventricular hypertrophy, however the signaling pathways that interact in these processes and are regulated by microRNAs inducing hypertension and the worsening of the disease still need to be elucidated. Thus, the aim of this review is to analyze the pattern of expression of microRNAs in these processes and the possible associated signaling pathways.

## 1. Introduction

Systemic arterial hypertension (SAH) is a multifactorial disease that is associated with genetic factors, such as inherited genes that favor increased blood pressure and environmental factors, including hypersodic and hypercaloric diet, overweight and obesity, physical inactivity, alcohol consumption in excess, advanced age, among other factors [1]. SAH is also defined as a chronic state of high blood pressure levels [2].

It is estimated that the global prevalence of SAH is greater than 30% in individuals aged 20 years or more. This rate varies when analyzed separately in some countries, with the percentage difference being almost 5% higher in medium- and low-income economy countries when compared to high-income economy countries [3]. In addition, it is known that the SAH is also associated to the development of type 2 diabetes and other comorbidities.

Several processes are associated with increased blood pressure levels and later with SAH, including: changes in the renin–angiotensin–aldosterone system (RAAS), activation of the sympathetic nervous system (SNS), endothelial dysfunction and increased oxidative stress [4]. These long-term pathophysiological processes result in hemodynamic pressure overload promoting cardiac remodeling and pathological cardiac hypertrophy in SAH [5].

Little is known, however, about the molecular mechanisms that initiate and govern these pathophysiological processes in SAH, which begin to be elucidated through microRNAs (miRNAs). MiRNAs are small ribonucleic acids (RNAs), non-coding RNA and gene expression regulators at the post-transcriptional level [6], their function is to degrade or inhibit one or several target messenger RNAs [7,8].

Some studies have reported miRNA deregulation in SAH, promoting activation of RAAS and SNS [9], and inducing pathological cardiac hypertrophy of the left ventricle [10]. However, the molecular mechanisms regulated by miRNAs that regulate the RAAS, SNS and left ventricular hypertrophy (LVH) in SAH still need to be elucidated. Thus, the aim of this review is to analyze the expression of deregulated miRNAs in RAAS, SNS and LVH in SAH, including possible signaling pathways associated with these processes.

## 2. MiRNAs and Activation of the Renin–Angiotensin–Aldosterone System in SAH

The renin–angiotensin–aldosterone system (RAAS) is of fundamental importance in the control of extravascular volume and blood pressure [11]. Due to its regulatory role, RAAS inhibition has therapeutic implications for several chronic diseases, such as SAH, type 2 diabetes and heart failure [12].

Renin is an enzyme synthesized by the juxtaglomerular cells of the kidney and stored in its inactive form as prorenin [13]. In situations of blood pressure drop, circulating volume depletion, reduced serum sodium concentration and sympathetic activation, prorenin molecules will be cleaved and renin will be released, which will act as the main regulator of RAAS [14].

Angiotensinogen is a circulating protein, synthesized by the liver, which will be metabolized under the action of renin, forming angiotensin I, a peptide. The conversion of angiotensin I (Ang-I) to angiotensin II (Ang-II) occurs mainly through the action of the angiotensin-converting enzyme (ACE) in the pulmonary microcirculation [15].

Angiotensin II acts by stimulating the type 1 angiotensin II (AT1R) receptor, causing vasoconstriction in the cardiovascular system, reducing the excretion of sodium and water, stimulating the synthesis of aldosterone, a mineralocorticoid synthesized in the glomerulosa of the adrenal cortex, among other functions [12,16]. Aldosterone also modulates circulating volume through marked stimulation of sodium reabsorption at the level of the renal tubules, with consequent fluid retention, in addition to potassium excretion [14,16].

RAAS imbalances contribute to the development of SAH, due to frequent and intense vasoconstriction of arterioles that cause an increase in peripheral vascular resistance and subsequently in blood pressure, in the long term, target organ damage occurs with more serious consequences [11,17].

Many miRNAs regulate RAAS genes. MiR-27b and miR-145 attenuate angiotensin converting enzyme-1 (ACE-1) expression, but these miRNAs are downregulated in hypertension, increasing ACE-1 expression [18,19]. MiR-143 and miR-421 are overexpressed in hypertension, targeting angiotensin converting enzyme-2 (ACE-2) and contributing to increased blood pressure [19,20].

MiR-483 targets multiple RAAS genes, such as angiotensinogen (AGT), ACE-1, ACE-2 and angiotensinogen receptor 2 (AGTR2), however, the expression of miR-483 is downregulated in vascular smooth muscle cells (VSMCs) stimulated with Ang-II, which leads to overexpression of its target genes and vascular hypertrophy [21]. MiR-143/145 cluster also regulates ACE-1, and when these miRNAs are poorly expressed in VSMCs, it promotes an increase in ACE-1 expression, inducing a reduction in its contractile phenotype and vascular dysfunction [22].

In this context, VSMCs stimulated with Ang-II present reduced miR-155 expression and increased AT1R expression, leading to increased cell proliferation, however, the increase in the expression of this miRNA can abrogate this effect [23]. Likewise, another miRNA, miR-130, was overexpressed, reducing the expression of the target gene GAX, contributing to the proliferation of VSMCs when stimulated with Ang-II [24]. MiR-27a also had its expression elevated in VSMCs stimulated with Ang-II, reducing the expression of α-smooth muscle-actin (α-SMA), leading to proliferation and migration of VSMCs, promoting change in contractile phenotype and reduction in vascular function [25], which favors an increase in peripheral vascular resistance and consequently induces an increase in blood pressure.

In Ang-II-stimulated rat aortic adventitial fibroblasts, miR-122 was overexpressed, decreasing the expression of sirtuin (SIRT-6), elabela (ELA) and ACE-2, promoting reduced autophagic flux and increased cell migration, oxidative stress, inflammation and apoptosis, leading to vascular remodeling [26]. Another miRNA that regulates the apoptosis process in hypertension is miR-133a. This miRNA targets the prorenin receptor (PRR), and in Ang-II-stimulated human umbilical vein endothelial cells (HUVECs) miR-133a was downregulated, increasing PRR expression, which will exacerbate the signaling pathway of the RAAS, promoting apoptosis [27].

In cardiac fibroblasts treated with Ang-II, miR-125b, miR-132 and miR-146b are overexpressed, inhibiting the target genes MMP9 and MMP16, while other miRNAs are downregulated, such as miR-181b, miR-204 and miR-300, increasing the expression of TIMP3 promoting fibrosis in these cells [28].

In another study with Ang-II-stimulated cardiac fibroblasts and HEK293N cells, four miRNAs were found to be overexpressed: miR-29b, miR-129, miR-132 and miR-212, activating Gαq/11, the extracellular regulated kinase1/2 (ERK-1/2) and AT1R, and the activation of this pathway can promote increased blood pressure [29]. MiR-132/212 cluster is overexpressed in the heart, aorta, kidney and blood circulation of Ang-II-induced hypertensive rats, as well as in the arteries of hypertensive patients, inducing endothelin receptor activation and cardiac hypertrophy [30]. It has also been shown that miR-132 regulates plasma renin levels by modulating blood pressure [31].

In hypertensive rats, miR-16 is overexpressed, reducing the expression of the vascular endothelial growth factor (VEGF), promoting an anti-angiogenic effect, and miR-21 is also highly expressed decreasing the expression of the anti-apoptotic Bcl-2, inducing apoptosis. On the other hand, in this same study, miR-126 is downregulated by increasing the expression of the phosphoinositol-3 kinase regulatory subunit 2 (PI3KR2) which negatively modulates the VEGFR, mitogen activated protein kinase (MAPK) and phosphoinositol-3 kinase (PI3K) signaling pathways, generating microvascular rarefaction in hypertension [32].

Microvascular rarefaction is a process that occurs in individuals with SAH that decreases blood flow in skeletal muscle capillaries, inducing an increase in total peripheral resistance, generating an increase in blood pressure. This process promotes changes in the expression of several miRNAs that regulate vascular remodeling in SAH, being an important factor to be analyzed in future studies for reverse modulation of these miRNAs in an attempt to attenuate this process [33].

Clinical studies also show changes in the expression of miRNAs in hypertensive patients. MiR-136 is downregulated in the serum of patients with hypertension and is associated with elevated levels of RAAS biochemical markers [34]. However, miR-202 is overexpressed in the blood of hypertensive patients, by reducing the receptor soluble (sST2), being associated with high levels of Ang-II and vascular inflammation [35].

Other miRNAs are also with high expression in hypertensive patients, such as miR-21, miR-126, miR-196a and miR-451, while others are with reduced expression, such as miR-181a, miR-638 and miR-663. It was even evidenced that miR-181a and miR-663 target the renin gene, demonstrating that these miRNAs potentially regulate blood pressure [36].

Another miRNA that regulates the renin gene is miR-25. This miRNA is downregulated in the serum of hypertensive patients, elevating the renin expression, promoting RAAS activation and consequently leading to hypertensive heart disease [37]. MiR-155 is also downregulated in the plasma of hypertensive patients, increasing AT1R expression, activating the RAAS signaling pathway, increasing blood pressure [38] (Table 1).

Thus, the RAAS signaling pathway is modulated by miRNAs to regulate blood pressure, however, many other miRNAs can regulate these same RAAS genes in SAH, but further studies are needed to confirm this hypothesis.

## 3. MiRNAs and Sympathetic Nervous System Activation in SAH

The sympathetic nervous system (SNS) plays a fundamental role in blood pressure control through regulatory mechanisms expressed based on the release of neurotransmitters (epinephrine, norepinephrine and dopamine) that act on vessels, kidneys and heart. Thus, changes in the functions of this system are related to the development of cardiovascular disorders [66].

Cardiac output and systemic vascular resistance are the main target components for the action of sympathetic neurotransmitters through their release, by central and reflex mechanisms, and binding to their adrenergic (α and β) and dopaminergic receptors. These mechanisms, when activated, promote an arteriolar vasoconstriction (through α-receptors) and an increase in cardiac output (through β-receptors) resulting in an elevation of blood pressure [67,68].

Activation of the SNS occurs in states of physical or emotional stress. However, in early stages of SAH, known as a hyperkinetic circulatory state, there is an increase in adrenergic impulse and a decrease in parasympathetic function [67], that is, there is an increase in plasma levels of sympathetic neurotransmitters concomitant with a loss of vagal inhibitory function. Furthermore, the magnitude of sympathetic activation parallels the severity of blood pressure elevation [68].

MiRNAs also participate in the regulation of the SNS. Specifically, miR-181a was downregulated in a genetic model of massive SNS activation, leading to increased renin expression, RAAS activation, and consequently hypertension in mice [49]. Overexpression of miR-135a and miR-376 were also associated with increased sympathetic nerve activity, contributing to exacerbated blood pressure and inflammation in spontaneously hypertensive rats [44].

In this context, miR-22 is also highly expressed in spontaneously hypertensive rats, reducing the expression of the Chromogranin A (CHGA), inducing greater central and peripheral nerve activity, contributing to the elevation of blood pressure [51]. A polymorphism in the CHGA 3’-untranslated region known as C+87T (rs7610), promotes increased inhibition of CHGA by miR-107, leading to increased sympathetic nerve activity, autonomic dysregulation, increased blood pressure and renal disease in a hypertensive mouse model [45].

Renal disease in hypertensive patients is very common due to increased renal sympathetic nervous system and this hyperactivation induces reduced expression of miR-133a [60]. This same study showed that the renal sympathetic denervation in these patients promotes increased expression of miR-133a, attenuating blood pressure, including being associated with decreased risk of developing hypertensive heart disease [60].

Other miRNAs, such as miR-200a, miR-200b, miR-205, miR-141, miR-192 and miR-429, are overexpressed in hypertensive patients with renal disease leading to nephrosclerosis, and the degree of elevation of these miRNAs was correlated with disease severity [69].

Furthermore, other miRNAs can be dysregulated due to activation of the sympathetic nervous system and inhibition of the parasympathetic nervous system in SAH, favoring the hemodynamic overload that generates long-term left ventricular hypertrophy.

## 4. MiRNAs and Left Ventricular Hypertrophy in SAH

Under physiological conditions, stroke volume is regulated by preload through mechanisms that involve the extension of cardiac fibers at the end of diastole associated with resistance imposed by afterload [70]. Mechanical stress on the heart induces changes involving strain (related to increased afterload) and shear stress (related to blood friction against the vessel wall) resulting in compensatory adaptive effects when chronically altered in order to keep the cardiac output [71].

This mechanical stress is one of the triggering factors of cardiac remodeling, which occurs through processes of cardiomyocyte hypertrophy, hyperplasia, hypertrophy of non-muscle cells and interstitial proliferation [72]. Cardiac cell remodeling and hypertrophy, in response to mechanical stress in SAH, are the mechanisms that lead to left ventricular hypertrophy (LVH) [73]. LVH in SAH induces an increase in muscle mass and myofibril growth in parallel, generating concentric hypertrophy, reducing the internal area of the cardiac chamber. Hyperplasia of vascular structures and collagen accumulation also occur, favoring cardiac fibrosis [74].

Several miRNAs participate in the LVH process. In human-induced pluripotent stem-cell-derived cardiomyocytes stimulated with endothelin-1 (ET-1), miR-19a, miR-21, miR-29b and miR-199 are overexpressed, generating cardiac hypertrophy in vitro, however, when the analysis was performed on circulating miRNAs in the serum of chagasic patients with LVH, only miR-19a, miR-21 and miR-29b were overexpressed [43]. In cardiomyocytes derived from neonatal rats stimulated with ET-1, miR-19a and miR-19b promoted hypertrophy of these cardiomyocytes, reducing the expression of anti-hypertrophic target genes atrogin-1 and muscle RING-finger protein-1 (MURF-1), and it also activated the pro-hypertrophic pathway calcineurin/nuclear factor of activated T-cells (NFAT) [41].

On the other hand, Ang-II-induced pressure overload in rats reduced the expression of miR-19a and miR-19b, increasing the expression of phosphodiesterase 5A (PDE5A), generating LVH, including, as the authors show in the same study, that a model of transgenic mouse overexpressing miR-19a and miR-19b reduced PDE5A expression, decreasing cardiac hypertrophy [55], demonstrating that an expression pattern response of miRNAs in cell culture can be quite different from the response in animal models.

MiR-21, miR-126 and miR-146 are overexpressed in a C576BJ mouse model of cardiac hypertrophy, while miR-29b, miR-133a, miR-133b, miR-149, miR-150 and miR-185 were downregulated after aortic binding constriction [46]. MiR-132 and miR-212 are also overexpressed, decreasing the expression of the FoxO3 transcription factor, inducing attenuation of autophagy and activation of the pro-hypertrophic calcineurin/NFAT signaling pathway, generating LVH, in both, in the culture of primary cardiomyocytes with different hypertrophic stimuli and in mice with transaortic constriction (TAC) and cardiac hypertrophy [40]. Likewise, in both in vitro and in vivo models stimulated with Ang-II, the expression of miR-410 and miR-495 is increased, modulating the expression of the hypertrophic genes Nppa and Nppb, promoting a robust hypertrophy of cardiomyocytes [48].

In an model of hypertensive mice overexpressing renin, it promoted SAH by increasing the expression of miR-208a and 208b, decreasing the expression of the transcription factor SOX-6, which is a repressor of alpha-myosin heavy chain (MyHC), increasing the expression of the latter gene, inducing LVH [47].

MiR-208b was also overexpressed in peripheral blood mononuclear cells from patients with SAH, in addition, other miRNAs were also overexpressed in these samples, such as miR-1, miR-21 and miR-499, while miR-26b and miR-133a were with reduced expression and this expression pattern correlated with LVH in these patients [58]. In studies in humans, animals and cells, the reduction of miR-133a expression promotes LVH or cardiomyocytes hypertrophy and the overexpression of this miRNA induces an anti-hypertrophic effect [75,76].

The miR-29 family (miR-29a, miR-29b and miR-29c), in turn, is overexpressed in the plasma of patients with SAH, regulating genes of the fibrotic and hypertrophic process, having a strong correlation with high blood pressure and LVH [61]. In agreement with these results, miR-29a was also shown with increased expression in the plasma of hypertensive patients with positive association with LVH [77].

On the other hand, miR-29b had its expression reduced in a mouse model of Ang-II-induced hypertension, increasing the expression of cholagen-1 (COL-I), transforming growth factor-β (TGF-β), α -SMA, tumor necrosis factor-α (TNF-α), interleukin-1β (IL-1β), with reduced expression of the mothers against decapentaplegic homolog 7 (SMAD7) gene, inducing LVH. However, the induction of SAMD7 overexpression prevented the loss of miR-29b, decreasing the expression of COL-I, TGF-β, α-SMA, TNF-α, IL-1β, reducing the inflammatory and fibrotic profile, in addition to attenuating the heart damage and LVH in these animals [54]. This finding demonstrates that the expression pattern of miRNAs may be different in hypertensive patients compared to animal models.

Another miRNA that is associated with the process of inflammation and cardiac hypertrophy is miR-155. In both in vivo and in vitro models of Ang-II-induced cardiac hypertrophy, miR-155 is overexpressed, reducing the expression of the inhibitor of nuclear factor kappa-B kinase subunit epsilon (IKBKE), promoting activation of the nuclear factor kappa- B (NF-κB), inflammation and cardiac hypertrophy [42]. In this same work, it was shown that the overexpression of the long non-coding RNA known as cytoskeleton regulator RNA (CYTOR), can serve as a sponge for miR-155, reducing the expression of this miRNA, and consecutively increasing the expression of IKBKE, inhibiting the NF-κB pathway, reducing inflammation and cardiomyocyte hypertrophy [42].

MiR-155 also had increased expression in the plasma of hypertensive patients, and this high expression was associated with inflammatory markers such as interleukin-6 (IL-6) and c-reactive protein [65]. Other circulating miRNAs also dysregulated in the plasma of hypertensive patients, such as miR-29 and 30a, are overexpressed, whereas miR-133 is downregulated [64], and these miRNAs are associated with the processes of inflammation and LVH [75,78].

**Figure 1:**
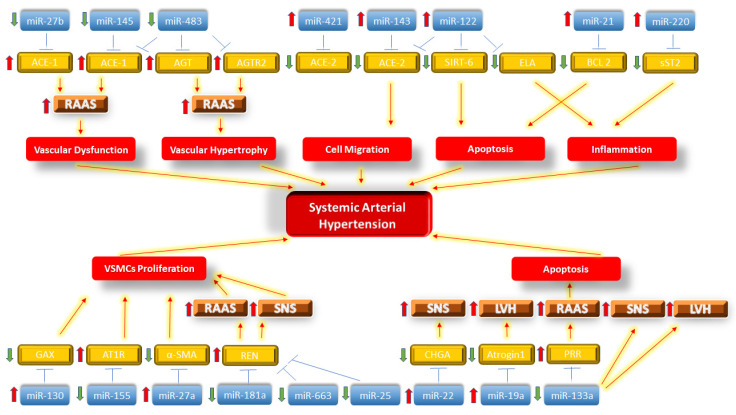
Schematic representation of miRNAs regulating genes and signaling pathways in RAAS, SNS and LVH in SAH. Thick red upward arrow: upregulation of miRNA or target gene; thick green downward arrow: downregulation of miRNA or target gene; blue thin arrow: indicates that miRNA regulates that gene; red thin arrow: indicates that the change in the signaling pathway promotes a specific outcome.

Many other miRNAs are regulating genes and signaling pathways in SAH, including other non-coding RNAs; the main miRNAs that regulate signaling pathways in the RAAS, SNS and LVH are shown in a schematic representation in (Figure 1). Furthermore, other epigenetic processes, such as DNA methylation, histone acetylation and deacetylation, are also being modulated by changing transcriptomics during the pressure overload process promoting SAH.

## 5. Overlapping miRNAs in RAAS, SNS and LVH in SAH

**Figure 2:**
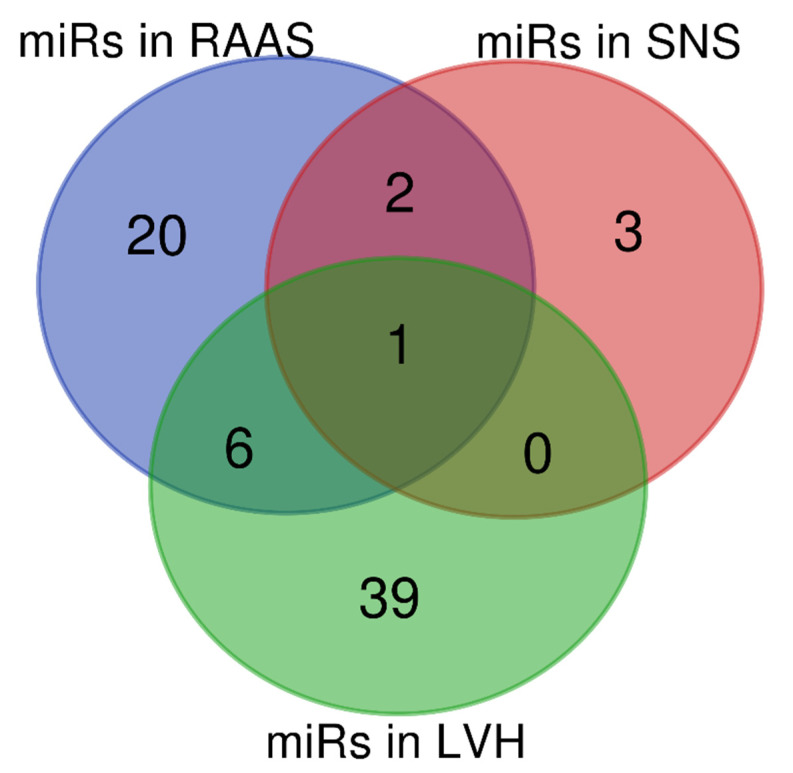
MiRNAs regulating RAAS, SNS and LVH.

After reviewing the expression of miRNAs in RAAS, SNS and LVH in in vitro, in vivo and clinical studies, despite different methodologies applied in these works, it was possible to identify six miRNAs (miR-21 miR-155 miR-132 miR-29b miR-126 miR-212) with altered expression regulating RAAS and LVH processes. It was also possible to identify two miRNAs (miR-181a miR-135a) modulating the RAAS and SNS processes. No miRNA was identified associated with the SNS and LVH processes. However, one miRNA was observed to regulate all processes, the miR-133a (Figure 2).

MiR-133a plays an important role in cardiac development and is known to regulate some cardiovascular diseases [79,80,81]. Here it was identified that miR-133a is involved in the regulation of three processes that are fundamental for the development of SAH and the clinical worsening of the disease. In this context, miR-133a is downregulated regulating signaling pathways in RAAS, SNS and LVH, as previously described. Interestingly, it was identified that miR-133a is regulating the PRR gene, activating the RAAS and SNS, promoting LVH, aggravating the disease. In addition, miR-133a is associated with other signaling pathways such as β-MHC and atrial natriuretic factor (ANF).

In addition to regulating these three overlapping processes, miR-133a also regulates vascular remodeling and fibrosis through the membrane type-1 matrix metalloproteinase (MT-1 MMP) target gene. MiR-133a was also reduced in aortic fibroblasts exposed to biaxial cyclic stretch, generating tension and vascular remodeling, including being downregulated in thoracic aortic tissue of Ang-II-induced hypertensive mice and in spontaneously hypertensive mice [82].

In situations of myocardial ischemia that occur in many hypertensive patients, miR-133a is also downregulated, promoting an increase in MT-1 MMP, in addition to elevating other pro-fibrotic genes such as transforming growth factor beta receptor 1 (TGFBR1), latent transforming growth factor binding protein 1 (LTBP1), matrix metalloproteinase 9 (MMP9), phosphorylated Smad2 (pSMAD2) and COLIAI, inducing activation of TGF-β signaling pathway, leading to vascular and cardiac fibrosis [83].

Furthermore, miR-133a was shown to be reduced in the left ventricle of rats with chronic administration of AngII, increasing the expression of the COLIAI gene, generating myocardial fibrosis [84]. All this evidence demonstrates that miR-133a plays a critical role in SAH, acting as a key element in this disease.

## 6. Conclusions

Several miRNAs are regulating target genes and altering signaling pathways in RAAS, SNS and LVH in SAH. Some of these miRNAs are associated and interact to activate these systems simultaneously and worsen the clinical status of these patients. Specifically, miR-133a has a key multiregulatory role in the three analyzed processes, regulating the PRR signaling pathway, and is also associated with other deleterious situations in SAH, such as cardiac fibrosis and myocardial ischemia. Thus, further studies are needed to analyze the expression of miRNAs in RAAS, SNS and LVH, and especially the activation of miR-133a as a potential therapy for inactivation of RAAS and SNS, inducing possible LVH attenuation in SAH.

## Figures and Tables

**Table 1:** Expression of miRNAs in RAAS, SNS and LVH.

MicroRNAs in RAAS, SNS and LVH				
In Vitro Studies				
MicroRNAs	Study Model	Findings	miRNA Targets	Reference
↓ miR-143/145	Vascular smooth muscle cells (VSMCs) obtained from miR143/145-/- mice	miR-143/145 deficiency reduces the contractility of vascular smooth muscle cells.	ACE	[22]
↓ miR-483-3p	VSMCs—human and rat aortic smooth muscle cells	miR483-3p is reduced after in vitro stimulation with angiotensin II, which activates the renin angiotensin aldosterone system (RAAS).	Multiple components of the RAS: ACE1, ACE2, AGTR2	[21]
↑ miR-130a	VSMCs were prepared from the thoracic aorta of Sprague–Dawley rats	miR-130a induces the proliferation of VSMCs, by targeting GAX, which has inhibitory actions on VSMCs proliferation.	GAX	[24]
↑ miR-124, miR-135a	HeLa cells	Mineralocorticoid receptor NR3C2 is a target of miR-124 and miR-135a, which can be involved in the regulation of RAAS.	NR3C2	[39]
↑ miR-29b, miR-129, miR-132, miR-212	Cardiac fibroblasts and HEK293N cells	Overexpressed miRNAs activate Gaq/11, ERK-1/2 and AT1R.	AT1R	[29]
↑ miR-132, miR-212	H9c2 cells and primary cardiomyocytes	MiRNAs 132 and 212 were overexpressed regulating FoxO3 inducing LVH.	FoxO3	[40]
↑ miR-125b, miR-132, miR-146b ↓ miR-181b, miR-204, miR-300	Cardiac fibroblasts treated by AngII	A group of dysregulated miRNAs when treated with AngII, demonstrating important roles in hypertension and cardiac fibrosis.	MMP9, MMP16, TIMP3	[28]
↓ miR-155	Primary VSMCs from the aorta of C57/BL6 mice	Angiotensin II stimulation decreases expression of miR-155, inducing cell proliferation and survival.	AT1R	[23]
↑ miR-19a, miR-19b	Cardiomyocytes derived from neonatal rats stimulated with ET-1	MiR-19a and miR-19 promoted cardiomyocyte hypertrophy by regulating atrogin-1 and MURF-1.	Atrogin-1, MURF-1	[41]
↑ miR-27a	VSMCs stimulated with Ang-II	MiR-27a was overexpressed generating proliferation, migration and vascular dysfunction.	aSMA	[25]
↓ miR-133a	HUVECs stimulated with Ang-II	MiR-133a was downregulated, increasing PRR expression, which will exacerbate the signaling pathway of the RAAS, promoting apoptosis.	PRR	[27]
↑ miR-155	Cardiomyocytes stimulated with Ang-II	MiR-155 was overexpressed reducing IKBKE promoting inflammation and cardiomyocyte hypertrophy.	IKBKE	[42]
↑ miR-19a, miR-21, miR-29b, miR-199b	Cardiac fibroblasts and ipsc-derived cardiomyocytes stimulated with ET-1	MiR-21 was overexpressed, leading to cardiac hypertrophy and fibrosis.	SPRY1	[43]
↑ miR-122	Rat aortic adventitial fibroblasts	MiR-122 was overexpressed, promoting reduced autophagic flux and increased cell migration, oxidative stress, inflammation and apoptosis.	SIRT-6, ELA, ACE2	[26]
**In Vivo Studies**				
↑ miR-135a, miR-376a	Spontaneous hypertensive rats	Downregulation of Agtrap transcript by miR-135a and miR-376a; disinhibition of AT1R signaling; miR-135a downregulates *Ptgr1* to increase the levels of LTB4, leading to the development of hypertension.	PTGR1, AGTRAP	[44]
↑ miR-107	Hypertensive mouse model	A polymorphism in the CHGA 3’-untranslated region known as C+87T (rs7610), promotes increased inhibition of CHGA by miR-107, leading to increased sympathetic nerve activity.	CHGA	[45]
↑ miR-21, miR-126, miR-146 ↓ miR-29b, miR-133a, miR-133b, miR-149, miR-150, miR-185	Cardiac hypertrophy C576BJ mice model	MicroRNAs were deregulated after aortic banding generating cardiac hypertrophy.	ANF, BNF, β-MHC	[46]
↑ miR-208a, miR-208b	Cardiac hyperaldosteronism (AS mice) and systemic hypertension (Ren)	Aldosterone and renin overexpression increases the expression of miR-208a and miR-208b inhibiting Sox6 and increasing cardiac hypertrophy.	Sox6	[47]
↑ miR-16, miR-21 ↓ miR-126	Spontaneous hypertensive rats	MiR-16 is overexpressed, reducing VEGF expression, promoting decreased angiogenesis and miR-21 is also highly expressed, attenuating Bcl2 expression, inducing apoptosis. miR-126 is downregulated increasing PI3KR2 expression by inhibiting the VEGFR pathway.	VEGF, Bcl2, PI3KR2	[32]
↑ miR-132, miR-212	Transaortic constriction mice (TAC)	MiRNAs 132 and 212 were overexpressed regulating FoxO3 inducing LVH.	FoxO3	[40]
↑ miR-132, miR-212	Angiotensin II-induced hypertensive rats	MiR-132/212 are increased in heart, kidney, aorta and plasma of angiotensin II-induced hypertensive rats.	PTEN, ERK/MAPK	[30]
↑ miR-410, miR-495	Ang-II stimulated rat model promoting cardiac hypertrophy	MiR-410 and miR-495 are increased in this cardiac hypertrophy model.	Nppa, Nppb	[48]
↓ miR-181a	Genetically hypertensive mice (BPH/2J)	miR-181a was downregulated by increasing REN expression, increasing sympathetic nervous system activity.	REN1	[49]
↓ miR-34b	Spontaneous hypertensive rats	miR-34b was found downregulated in spontaneous hypertensive rats, increasing the levels of CDK6, leading to increased proliferation of VSMCs.	CDK6	[50]
↑ miR-22	Spontaneous hypertensives rats	miR-22 associated with dysregulation of Chga in brainstem cardiovascular control nuclei contributing to the pathogenesis of hypertension in SHR.	Chga	[51]
↑ miR-153	Spontaneous hypertensives rats	miR-153 upregulation leads to reduced Kv7.4 channel expression, vasoconstriction, and vascular wall thickening.	KCNQ4, Kv7.4	[52]
↑ miR-487b	Rat model of angiotensin II-induced hypertension	MiR-487b is upregulated by AngII and targets the vasoactive molecule IRS1, causing loss of adventitial and medial integrity.	IRS1	[53]
↓ miR-29b	Mouse model of Ang II-induced hypertension	MiR-29b is downregulated in mouse model of Ang-II-induced hypertension, promoting LVH.	COL-I, TGFb	[54]
↓ miR-19a, miR-19b	Ang-II-induced cardiac hypertrophy mouse model	Ang-II-induced pressure overload in rats reduced the expression of miR-19a and miR-19b, increasing the expression of PDE5A, generating LVH.	PDE5A	[55]
↑ miR-21	ALDO/SALT Hypertensives Mice	This study showed that miR-21 is upregulated by excess ALDO in the LV.	Spry1, Spry2, PTEN, PDCD4, Bcl2	[56]
**Clinical Studies**				
↑ miR-92a	Hypertensive patients (n = 240)	Plasma levels of miR-92a are increased in hypertensive patients and correlate with 24 h mean systolic and diastolic blood pressure, 24 h mean pulse pressure, 24 h daytime and nighttime pulse pressure, increased carotid intima–media thickness and carotid-femoral pulse wave velocity.	KLF2, KLF4, eNOS	[57]
↑ miR-1, miR-208b, miR-499, miR-21 ↓ miR-133a, miR-26b	Hypertensive patients (n = 132)	Analysis of expression in PBMCs: miR-1, miR-133a, miR-26b, miR-208b, miR-499, and miR-21 show distinct expression profiles in hypertensive patients compared to healthy subjects; association with LVH.	MEF2a, BMPR2, PDCD4, PTEN	[58]
↑ miR-505	Hypertensive patients (n = 192)	Plasma levels of miR-505 are increased in hypertensive patients compared to healthy subjects and is positively correlated with systolic blood pressure; impaired endothelial migration and tube formation in culture by direct regulation of FGF18 and indirect regulation of HMGB1.	FGF18	[59]
↓ miR-133a	Hypertensive patients (n = 90)	Increased renal sympathetic nervous system induces downregulation of miR-133a.	PRR	[60]
↑ miR-202	Hypertensive patients (n = 182)	miR-202-3p exerts a protective role against EH by antagonizing the induction of sST2 by Ang-II.	ST2	[35]
↑ miR-29a, miR-29b, miR-29c	Hypertensive patients (n = 84)	Plasma levels of mir-29a, b and c were increased in patients with hypertension, with positive correlations with office systolic and diastolic blood pressure, office pulse pressure, 24 h mean systolic and diastolic blood pressure, 24 h mean pulse pressure and left ventricular hypertrophy.	COL1A1, COL1A2, COL3A1, VEGFA, TGF-β	[61]
↓ miR-136	Hypertensive patients (n = 110)	miR-136 is downregulated in patients with hypertension and is associated with elevated levels of RAAS biochemical markers.	Wnt, Notch3	[34]
↑ miR-516b, miR-600, miR-605, miR-623, let-7e ↓ miR-18b, miR-30d, miR-296-5p, miR-324-3p, miR-486-5p, miR-518b, miR-1236, miR-1227	Hypertensive patients (n = 194)	Plasma levels of miRNAs were distinct plasma miRNA expression pattern in hypertensive patients, compared with healthy subjects.	MAPK10, RICTOR, NFAT5, MAP3K9, MAP3K1, STAT3	[62]
↑ miR-132, miR-212	Hypertensive patients (n = 64)	miR-132/212 are increased in the arteries of hypertensive patients.	PTEN, ERK/MAPK	[30]
↓ miR-126	Hypertensive patients (n = 89)	Hypertensive patients showed significantly lower miR-126 expression levels in PBMCs, and positive correlation with 24 h mean pulse pressure.	SPRED-1, VEGF, PI3KR2	[63]
↓ miR-155	Hypertensive patients (n = 64)	AT1R protein expression was positively correlated with systolic and diastolic blood pressure and negatively correlated with miR-155 expression level in PBMCs.	AT1R	[38]
↑ miR-21, miR-126, miR-196a, miR-451 ↓ miR-181a, miR-638, miR-663	Hypertensive patients (n = 14)	MiR-663 can regulate REN and APOE mRNA levels, whereas miR-181a regulated REN and AIFM1 mRNA.	REN, APOE, AIFM1	[36]
↑ miR-29, miR-30a ↓ miR-133	Hypertensive patients	MiRNAs are dysregulated in the plasma of hypertensive patients, associated with cardiomyocyte hypertrophy.	TGF-β1, Sp-1	[64]
↓ miR-25	Hypertensive heart disease patients	miR-25 is downregulated in the serum of hypertensive patients, elevating the renin expression, promoting RAAS activation.	REN	[37]
↑ miR-155	Hypertensive patients	MiR-155 is overexpressed and associated with inflammatory markers.	TGF-β1	[65]

## References

[1] Oparil S., Acelajado M.C., Bakris G.L., Berlowitz D.R., Cífková R., Dominiczak A.F., Grassi G., Jordan J., Poulter N.R., Rodgers A. (2018). Hypertension. Nat. Rev. Dis. Prim..

[2] Neves V.J., Fernandes T., Roque F.R., Soci U.P., Melo S.F., de Oliveira E.M. (2014). Exercise training in hypertension: Role of microRNAs. World J. Cardiol..

[3] Mills K.T., Bundy J.D., Kelly T.N., Reed J.E., Kearney P.M., Reynolds K., Chen J., He J. (2016). Global Disparities of Hypertension Prevalence and Control: A Systematic Analysis of Population-based Studies from 90 Countries. Circulation.

[4] Klimczak D., Jazdzewski K., Kuch M. (2017). Regulatory mechanisms in arterial hypertension: Role of microRNA in pathophysiology and therapy. Blood Press..

[5] Frieler R.A., Mortensen R.M. (2015). Immune Cell and Other Non-Cardiomyocyte Regulation of Cardiac Hypertrophy and Remodeling. Circulation.

[6] Improta-Caria A.C., Nonaka C.K.V., Cavalcante B.R.R., De Sousa R.A.L., Júnior R.A., de Souza B.S.F. (2020). Modulation of microRNAs as a potential molecular mechanism involved in the beneficial actions of physical exercise in Alzheimer disease. Int. J. Mol. Sci..

[7] Baek D., Villén J., Shin C., Camargo F.D., Gygi S.P., Bartel D.P. (2008). The impact of microRNAs on protein output. Nature.

[8] Improta-Caria A.C., Aras R. (2021). Treinamento com Exercício Físico e Doença de Chagas: Função Potencial dos MicroRNAs. Arq. Bras. Cardiol..

[9] Caria A.C.I., Nonaka C.K.V., Pereira C.S., Soares M.B.P., Macambira S.G., Souza B.S.D.F. (2018). Exercise training-induced changes in microRNAs: Beneficial regulatory effects in hypertension, type 2 diabetes, and obesity. Int. J. Mol. Sci..

[10] Watanabe K., Narumi T., Watanabe T., Otaki Y., Takahashi T., Aono T., Goto J., Toshima T., Sugai T., Wanezaki M. (2020). The association between microRNA-21 and hypertension-induced cardiac remodeling. PLoS ONE.

[11] Patel S., Rauf A., Khan H., Abu-Izneid T. (2017). Renin-angiotensin-aldosterone (RAAS): The ubiquitous system for homeostasis and pathologies. Biomed. Pharmacother..

[12] Mirabito Colafella K.M., Bovée D.M., Danser A.H.J. (2019). The renin-angiotensin-aldosterone system and its therapeutic targets. Exp. Eye Res..

[13] Persson P.B. (2003). Renin: Origin, secretion and synthesis. J. Physiol..

[14] Muñoz-Durango N., Fuentes C.A., Castillo A.E., González-Gómez L.M., Vecchiola A., Fardella C.E., Kalergis A.M. (2016). Role of the Renin-Angiotensin-Aldosterone System beyond Blood Pressure Regulation: Molecular and Cellular Mechanisms Involved in End-Organ Damage during Arterial Hypertension. Int. J. Mol. Sci..

[15] Chappell M.C. (2016). Biochemical evaluation of the renin-angiotensin system: The good, bad, and absolute?. Am. J. Physiol.-Heart Circ. Physiol..

[16] Gorini S., Kim S.K., Infante M., Mammi C., La Vignera S., Fabbri A., Jaffe I.Z., Caprio M. (2019). Role of Aldosterone and Mineralocorticoid Receptor in Cardiovascular Aging. Front. Endocrinol..

[17] Takahashi H., Yoshika M., Komiyama Y., Nishimura M. (2011). The central mechanism underlying hypertension: A review of the roles of sodium ions, epithelial sodium channels, the renin–angiotensin–aldosterone system, oxidative stress and endogenous digitalis in the brain. Hypertens. Res..

[18] Hu B., Song J.T., Qu H.Y., Bi C.L., Huang X.Z., Liu X.X., Zhang M. (2014). Mechanical Stretch Suppresses microRNA-145 Expression by Activating Extracellular Signal-Regulated Kinase 1/2 and Upregulating Angiotensin-Converting Enzyme to Alter Vascular Smooth Muscle Cell Phenotype. PLoS ONE.

[19] Chen L.J., Xu R., Yu H.M., Chang Q., Zhong J.C. (2015). The ACE2/apelin signaling, microRNAs, and hypertension. Int. J. Hypertens..

[20] Lambert D.W., Lambert L.A., Clarke N.E., Hooper N.M., Porter K.E., Turner A.J. (2014). Angiotensin-converting enzyme 2 is subject to post-transcriptional regulation by miR-421. Clin. Sci..

[21] Kemp J.R., Unal H., Desnoyer R., Yue H., Bhatnagar A., Karnik S.S. (2014). Angiotensin II-regulated microRNA 483-3p directly targets multiple components of the renin-angiotensin system. J. Mol. Cell. Cardiol..

[22] Boettger T., Beetz N., Kostin S., Schneider J., Krüger M., Hein L., Braun T. (2009). Acquisition of the contractile phenotype by murine arterial smooth muscle cells depends on the Mir143/145 gene cluster. J. Clin. Investig..

[23] Yang L., Liu G., Zhu G., Liu H., Guo R., Qi F., Zou J. (2014). MicroRNA-155 inhibits angiotensin II-induced vascular smooth muscle cell proliferation. J. Renin. Angiotensin. Aldosterone. Syst..

[24] Wu W.-H., Hu C.-P., Chen X.-P., Zhang W.-F., Li X.-W., Xiong X.-M., Li Y.-J. (2011). MicroRNA-130a mediates proliferation of vascular smooth muscle cells in hypertension. Am. J. Hypertens..

[25] Xu M., Deng H., Li H. (2019). MicroRNA-27a regulates angiotensin II-induced vascular smooth muscle cell proliferation and migration by targeting α-smooth muscle-actin in vitro. Biochem. Biophys. Res. Commun..

[26] Song J., Yang M., Liu Y., Song J., Wang J., Chi H., Liu X., Zuo K., Yang X., Zhong J. (2020). MicroRNA-122 aggravates angiotensin II-mediated apoptosis and autophagy imbalance in rat aortic adventitial fibroblasts via the modulation of SIRT6-elabela-ACE2 signaling. Eur. J. Pharmacol..

[27] Liu B., Lan M., Wei H., Zhang D., Liu J., Teng J. (2019). Downregulated microRNA-133a induces HUVECs injury: Potential role of the (pro) renin receptor in angiotensin II-dependent hypertension. Mol. Med. Rep..

[28] Jiang X., Ning Q., Wang J. (2013). Angiotensin II induced differentially expressed microRNAs in adult rat cardiac fibroblasts. J. Physiol. Sci..

[29] Jeppesen P.L., Christensen G.L., Schneider M., Nossent A.Y., Jensen H.B., Andersen D.C., Eskildsen T., Gammeltoft S., Hansen J.L., Sheikh S.P. (2011). Angiotensin II type 1 receptor signalling regulates microRNA differentially in cardiac fibroblasts and myocytes. Br. J. Pharmacol..

[30] Eskildsen T.V., Jeppesen P.L., Schneider M., Nossent A.Y., Sandberg M.B., Hansen P.B.L., Jensen C.H., Hansen M.L., Marcussen N., Rasmussen L.M. (2013). Angiotensin II regulates microRNA-132/-212 in hypertensive rats and humans. Int. J. Mol. Sci..

[31] Jan Van Zonneveld A., Au Y.W., Stam W., Van Gelderen S., Rotmans J.I., Deen P.M.T., Rabelink T.J., Bijkerk R. (2020). MicroRNA-132 regulates salt-dependent steady-state renin levels in mice. Commun. Biol..

[32] Fernandes T., Magalhães F.C., Roque F.R., Phillips M.I., Oliveira E.M. (2012). Exercise training prevents the microvascular rarefaction in hypertension balancing angiogenic and apoptotic factors: Role of microRNAs-16, -21, and -126. Hypertension.

[33] Shi L., Liao J., Liu B., Zeng F., Zhang L. (2015). Mechanisms and therapeutic potential of microRNAs in hypertension. Drug Discov. Today.

[34] Chu H.T., Li L., Jia M., Diao L.L., Li Z.B. (2020). Correlation between serum microRNA-136 levels and RAAS biochemical markers in patients with essential hypertension. Eur. Rev. Med. Pharmacol. Sci..

[35] Li L., Zhong D., Xie Y., Yang X., Yu Z., Zhang D., Jiang X., Wu Y., Wu F. (2020). Blood microRNA 202-3p associates with the risk of essential hypertension by targeting soluble ST2. Biosci. Rep..

[36] Marques F.Z., Campain A.E., Tomaszewski M., Zukowska-Szczechowska E., Yang Y.H.J., Charchar F.J., Morris B.J. (2011). Gene Expression Profiling Reveals Renin mRNA Overexpression in Human Hypertensive Kidneys and a Role for MicroRNAs. Hypertension.

[37] Li H., Xie Y., Liu Y., Qi Y., Tang C., Li X., Zuo K., Sun D., Shen Y., Pang D. (2018). Alteration in microRNA-25 expression regulate cardiac function via renin secretion. Exp. Cell Res..

[38] Ceolotto G., Papparella I., Bortoluzzi A., Strapazzon G., Ragazzo F., Bratti P., Fabricio A.S., Squarcina E., Gion M., Palatini P. (2011). Interplay between miR-155, AT1R A1166C polymorphism, and AT1R expression in young untreated hypertensives. Am. J. Hypertens..

[39] Sõber S., Laan M., Annilo T. (2010). MicroRNAs miR-124 and miR-135a are potential regulators of the mineralocorticoid receptor gene (NR3C2) expression. Biochem. Biophys. Res. Commun..

[40] Ucar A., Gupta S.K., Fiedler J., Erikci E., Kardasinski M., Batkai S., Dangwal S., Kumarswamy R., Bang C., Holzmann A. (2012). The miRNA-212/132 family regulates both cardiac hypertrophy and cardiomyocyte autophagy. Nat. Commun..

[41] Song D.W., Ryu J.Y., Kim J.O., Kwon E.J., Kim D.H. (2014). The miR-19a/b family positively regulates cardiomyocyte hypertrophy by targeting atrogin-1 and MuRF-1. Biochem. J..

[42] Yuan Y., Wang J., Chen Q., Wu Q., Deng W., Zhou H., Shen D. (2019). Long non-coding RNA cytoskeleton regulator RNA (CYTOR) modulates pathological cardiac hypertrophy through miR-155-mediated IKKi signaling. Biochim. Biophys. Acta-Mol. Basis Dis..

[43] Nonaka C.K.V., Macêdo C.T., Cavalcante B.R.R., De Alcântara A.C., Silva D.N., Bezerra M.D.R., Caria A.C.I., Tavora F.R.F., Neto J.D.D.S., Noya-Rabelo M.M. (2019). Circulating miRNAs as potential biomarkers associated with cardiac remodeling and fibrosis in chagas disease cardiomyopathy. Int. J. Mol. Sci..

[44] DeCicco D., Zhu H., Brureau A., Schwaber J.S., Vadigepalli R. (2015). MicroRNA network changes in the brain stem underlie the development of hypertension. Physiol. Genom..

[45] Zhang K., Mir S.A., Makena Hightower C., Miramontes-Gonzalez J.P., Maihofer A.X., Chen Y., Mahata S.K., Nievergelt C.M., Schork N.J., Freedman B.I. (2015). Molecular mechanism for hypertensive renal disease: Differential regulation of chromogranin a expression at 3′-untranslated region polymorphism C+87T by MicroRNA-107. J. Am. Soc. Nephrol..

[46] Cheng Y., Ji R., Yue J., Yang J., Liu X., Chen H., Dean D.B., Zhang C. (2007). MicroRNAs are aberrantly expressed in hypertrophic heart: Do they play a role in cardiac hypertrophy?. Am. J. Pathol..

[47] Azibani F., Devaux Y., Coutance G., Schlossarek S., Polidano E., Fazal L., Merval R., Carrier L., Solal A.C., Chatziantoniou C. (2012). Aldosterone inhibits the fetal program and increases hypertrophy in the heart of hypertensive mice. PLoS ONE.

[48] Clark A.L., Maruyama S., Sano S., Accorsi A., Girgenrath M., Walsh K., Naya F.J. (2016). miR-410 and miR-495 Are Dynamically Regulated in Diverse Cardiomyopathies and Their Inhibition Attenuates Pathological Hypertrophy. PLoS ONE.

[49] Jackson K.L., Gueguen C., Lim K., Eikelis N., Stevenson E.R., Charchar F.J., Lambert G.W., Burke S.L., Paterson M.R., Marques F.Z. (2020). Neural suppression of miRNA-181a in the kidney elevates renin expression and exacerbates hypertension in Schlager mice. Hypertens. Res..

[50] Yang F., Li H., Du Y., Shi Q., Zhao L. (2017). Downregulation of microRNA-34b is responsible for the elevation of blood pressure in spontaneously hypertensive rats. Mol. Med. Rep..

[51] Friese R.S., Altshuler A.E., Zhang K., Miramontes-Gonzalez J.P., Hightower C.M., Jirout M.L., Salem R.M., Gayen J.R., Mahapatra N.R., Biswas N. (2013). MicroRNA-22 and promoter motif polymorphisms at the Chga locus in genetic hypertension: Functional and therapeutic implications for gene expression and the pathogenesis of hypertension. Hum. Mol. Genet..

[52] Carr G., Barrese V., Stott J.B., Povstyan O.V., Jepps T.A., Figueiredo H.B., Zheng D., Jamshidi Y., Greenwood I.A. (2016). MicroRNA-153 targeting of KCNQ4 contributes to vascular dysfunction in hypertension. Cardiovasc. Res..

[53] Nossent A.Y., Eskildsen T.V., Andersen L.B., Bie P., Brønnum H., Schneider M., Andersen D.C., Welten S.M., Jeppesen P.L., Hamming J.F. (2013). The 14q32 microRNA-487b targets the antiapoptotic insulin receptor substrate 1 in hypertension-induced remodeling of the aorta. Ann. Surg..

[54] Wei L.H., Huang X.R., Zhang Y., Li Y.Q., Chen H.Y., Yan B.P., Yu C.-M., Lan H.Y. (2013). Smad7 inhibits angiotensin II-induced hypertensive cardiac remodelling. Cardiovasc. Res..

[55] Liu K., Hao Q., Wei J., Li G.H., Wu Y., Zhao Y.F. (2018). MicroRNA-19a/b-3p protect the heart from hypertension-induced pathological cardiac hypertrophy through PDE5A. J. Hypertens..

[56] Syed M., Ball J.P., Mathis K.W., Hall M.E., Ryan M.J., Rothenberg M.E., Yanes Cardozo L.L., Romero D.G. (2018). Microrna-21 ablation exacerbates aldosterone-mediated cardiac injury, remodeling, and dysfunction. Am. J. Physiol.-Endocrinol. Metab..

[57] Huang Y., Tang S., Ji-yan C., Huang C., Li J., Cai A., Feng Y. (2017). Circulating miR-92a expression level in patients with essential hypertension: A potential marker of atherosclerosis. J. Hum. Hypertens..

[58] Kontaraki J.E., Marketou M.E., Parthenakis F.I., Maragkoudakis S., Zacharis E.A., Petousis S., Kochiadakis G.E., Vardas P.E. (2015). Hypertrophic and antihypertrophic microRNA levels in peripheral blood mononuclear cells and their relationship to left ventricular hypertrophy in patients with essential hypertension. J. Am. Soc. Hypertens..

[59] Yang Q., Jia C., Wang P., Xiong M., Cui J., Li L., Wang W., Wu Q., Chen Y., Zhang T. (2014). MicroRNA-505 identified from patients with essential hypertension impairs endothelial cell migration and tube formation. Int. J. Cardiol..

[60] Dörr O., Liebetrau C., Möllmann H., Gaede L., Troidl C., Lankes S., Guckel D., Boeder N., Voss S., Bauer T. (2016). Effect of Renal Sympathetic Denervation on Specific MicroRNAs as an Indicator of Reverse Remodeling Processes in Hypertensive Heart Disease. J. Clin. Hypertens..

[61] Huang Y., Tang S., Huang C., Chen J., Li J., Cai A., Feng Y. (2017). Circulating miRNA29 family expression levels in patients with essential hypertension as potential markers for left ventricular hypertrophy. Clin. Exp. Hypertens..

[62] Li S., Zhu J., Zhang W., Chen Y., Zhang K., Popescu L.M., Ma X., Bond Lau W., Rong R., Yu X. (2011). Signature microRNA Expression Profile of Essential Hypertension and Its Novel Link to Human Cytomegalovirus Infection. Circulation.

[63] Kontaraki J.E., Marketou M.E., Zacharis E.A., Parthenakis F.I., Vardas P.E. (2014). Differential expression of vascular smooth muscle-modulating microRNAs in human peripheral blood mononuclear cells: Novel targets in essential hypertension. J. Hum. Hypertens..

[64] Huang Y.Q., Huang C., Chen J.Y., Li J., Feng Y.Q. (2016). The association of circulating miR-30a, miR-29 and miR-133 with white-coat hypertension. Biomark. Med..

[65] Huang Y.Q., Huang C., Zhang B., Feng Y.Q. (2020). Association of circulating miR-155 expression level and inflammatory markers with white coat hypertension. J. Hum. Hypertens..

[66] DeLalio L.J., Sved A.F., Stocker S.D. (2020). Sympathetic Nervous System Contributions to Hypertension: Updates and Therapeutic Relevance. Can. J. Cardiol..

[67] Grassi G., Ram V.S. (2016). Evidence for a critical role of the sympathetic nervous system in hypertension. J. Am. Soc. Hypertens..

[68] Seravalle G., Mancia G., Grassi G. (2014). Role of the Sympathetic Nervous System in Hypertension and Hypertension-Related Cardiovascular Disease. High Blood Press. Cardiovasc. Prev..

[69] Wang G., Kwan B.C.-H., Lai F.M.-M., Choi P.C.-L., Chow K.-M., Li P.K.-T., Szeto C.-C. (2010). Intrarenal Expression of miRNAs in Patients with Hypertensive Nephrosclerosis. Am. J. Hypertens..

[70] Stansfield W.E., Ranek M., Pendse A., Schisler J.C., Wang S., Pulinilkunnil T., Willis M.S. (2014). The Pathophysiology of Cardiac Hypertrophy and Heart Failure. Cell. Mol. Pathobiol. Cardiovasc. Dis..

[71] Stevens S.M., Reinier K., Chugh S.S. (2013). Increased Left Ventricular Mass as a Predictor of Sudden Cardiac Death: Is it Time to put it to the Test?. Circ. Arrhythm. Electrophysiol..

[72] Nadruz W. (2014). Myocardial remodeling in hypertension. J. Hum. Hypertens..

[73] Shenasa M., Shenasa H. (2017). Hypertension, left ventricular hypertrophy, and sudden cardiac death. Int. J. Cardiol..

[74] Cacciapuoti F. (2011). Molecular mechanisms of left ventricular hypertrophy (LVH) in systemic hypertension (SH)—possible therapeutic perspectives. J. Am. Soc. Hypertens..

[75] Carè A., Catalucci D., Felicetti F., Bonci D., Addario A., Gallo P., Bang M.-L., Segnalini P., Gu Y., Dalton N.D. (2007). MicroRNA-133 controls cardiac hypertrophy. Nat. Med..

[76] Matkovich S.J., Wang W., Tu Y., Eschenbacher W.H., Dorn L.E., Condorelli G., Diwan A., Nerbonne J.M., Dorn G.W. (2010). MicroRNA-133a protects against myocardial fibrosis and modulates electrical repolarization without affecting hypertrophy in pressure-overloaded adult hearts. Circ. Res..

[77] Yao S.Y., Liu J., Li Y., Wang M., Wang C., Xue H. (2019). Association between plasma microRNA-29a and left ventricular hypertrophy in patients with hypertension. Zhonghua Xin Xue Guan Bing Za Zhi.

[78] Zhang Y., Cai S., Ding X., Lu C., Wu R., Wu H., Shang Y., Pang M. (2021). MicroRNA-30a-5p silencing polarizes macrophages toward M2 phenotype to alleviate cardiac injury following viral myocarditis by targeting SOCS1. Am. J. Physiol. Heart Circ. Physiol..

[79] Xiao Y., Zhao J., Tuazon J.P., Borlongan C.V., Yu G. (2019). MicroRNA-133a and Myocardial Infarction. Cell Transplant..

[80] Hromadnikova I., Kotlabova K., Dvorakova L., Krofta L. (2020). Diabetes Mellitus and Cardiovascular Risk Assessment in Mothers with a History of Gestational Diabetes Mellitus Based on Postpartal Expression Profile of MicroRNAs Associated with Diabetes Mellitus and Cardiovascular and Cerebrovascular Diseases. Int. J. Mol. Sci..

[81] Renaud L., Harris L.G., Mani S.K., Kasiganesan H., Chou J.C., Baicu C.F., Van Laer A., Akerman A.W., Stroud R.E., Jones J.A. (2015). HDACs Regulate miR-133a Expression in Pressure Overload Induced Cardiac Fibrosis. Circ. Heart Fail..

[82] Akerman A.W., Blanding W.M., Stroud R.E., Nadeau E.K., Mukherjee R., Ruddy J.M., Zile M.R., Ikonomidis J.S., Jones J.A. (2019). Elevated Wall Tension Leads to Reduced miR-133a in the Thoracic Aorta by Exosome Release. J. Am. Heart Assoc. Cardiovasc. Cerebrovasc. Dis..

[83] Eckhouse S.R., Akerman A.W., Logdon C.B., Oelsen J.M., O’Quinn E.C., Nadeau E.K., Stroud R.E., Mukherjee R., Jones J.A., Spinale F.G. (2013). Differential membrane type 1 matrix metalloproteinase substrate processing with ischemia–reperfusion: Relationship to interstitial microRNA dynamics and myocardial function. J. Thorac. Cardiovasc. Surg..

[84] Castoldi G., di Gioia C.R.T., Bombardi C., Catalucci D., Corradi B., Gualazzi M.G., Leopizzi M., Mancini M., Zerbini G., Condorelli G. (2012). MiR-133a regulates collagen 1A1: Potential role of miR-133a in myocardial fibrosis in angiotensin II-dependent hypertension. J. Cell. Physiol..

